# Cationic liposomes for generic signal amplification strategies in bioassays

**DOI:** 10.1007/s00216-020-02612-w

**Published:** 2020-04-06

**Authors:** Carola Hofmann, Barbara Kaiser, Susanne Maerkl, Axel Duerkop, Antje J. Baeumner

**Affiliations:** Institute of Analytical Chemistry, Chemo- and Biosensors, University of Regensburg, Universitätsstraße 31, 93053 Regensburg, Germany

**Keywords:** Liposomes, Electrostatic interaction, Bioanalysis, Bacteria, *E. coli*

## Abstract

Liposomes have been widely applied in bioanalytical assays. Most liposomes used bare negative charges to prevent non-specific binding and increase colloidal stability. Here, in contrast, highly stable, positively charged liposomes entrapping the fluorescent dye sulforhodamine B (SRB) were developed to serve as a secondary, non-specific label‚ and signal amplification tool in bioanalytical systems by exploiting their electrostatic interaction with negatively charged vesicles, surfaces, and microorganisms. The cationic liposomes were optimized for long-term stability (> 5 months) and high dye entrapment yield. Their capability as secondary, non-specific labels was first successfully proven through electrostatic interactions of cationic and anionic liposomes using dynamic light scattering, and then in a bioassay with fluorescence detection leading to an enhancement factor of 8.5 without any additional surface blocking steps. Moreover, the cationic liposomes bound efficiently to anionic magnetic beads were stable throughout magnetic separation procedures and could hence serve directly as labels in magnetic separation and purification strategies. Finally, the electrostatic interaction was exploited for the direct, simple, non-specific labeling of gram-negative bacteria. Isolated *Escherichia coli* cells were chosen as models and direct detection was demonstrated via fluorescent and chemiluminescent liposomes. Thus, these cationic liposomes can be used as generic labels for the development of ultrasensitive bioassays based on electrostatic interaction without the need for additional expensive recognition units like antibodies, where desired specificity is already afforded through other strategies.

**Electronic supplementary material:**

The online version of this article (10.1007/s00216-020-02612-w) contains supplementary material, which is available to authorized users.

**Figure:**
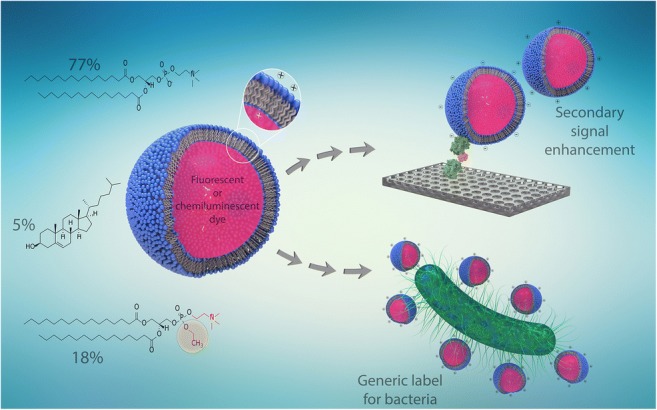
Graphical abstract

## Introduction

The labeling of biomolecules such as proteins, DNA, or microorganisms is often indispensable in bioanalysis [1–3]. This can be applied for the visualization of the structure or function of the molecules, and in some cases, it also enables their identification and quantitative analysis [3, 4]. There are a variety of different labels on the market which include, e.g., several dyes [4, 5], enzymes [6–8], radiolabels [9, 10], or nanoparticles [11–13]. In all cases, a sufficient amount of label is necessary to achieve the required contrast and sensitivity. Therefore, also signal amplification strategies play an important role which is often achieved using nanomaterials [14–16]. In the case of liposomes, this can be achieved via the entrapment of numerous signaling molecules inside the vesicles which leads to a significant increase in the signal upon their release [7, 17–19]. Moreover, the attachment of receptors, like antibodies or aptamers, on the vesicle surface enables a specific recognition of the biomolecule of interest [17]. This renders liposomes a powerful and versatile label in bioanalysis. However, especially the need for an additional labeling of the liposome surface with specific receptors significantly increases the cost of the vesicle preparation. Furthermore, it makes the preparation more time-consuming as it often requires an additional step for the covalent functionalization and purification post synthesis [20].

Therefore, strategies that omit such an additional labeling have been proposed which often include the use of polydiacetylene (PDA) vesicles [17]. These vesicles respond to changes in their environment, like temperature or pH, via conformational changes of the conjugated backbone [21, 22]. They have, e.g., been reported in immunosensors [22], for the detection of cationic surfactants [21] or of pathogenic bacteria [23]. Here, e.g., amine-functionalized PDA vesicles have been applied which respond to bacteria that secrete the negatively charged surfactin [23]. Liposome-based assays without specific receptors can rely on the cleavage of the membrane via enzymes like phospholipase [24] or sphingomyelinase [25]. In addition, also the surface charge of the bacteria themselves may be exploited for recognition. Bacteria usually exhibit an anionic surface charge caused by the thick negatively charged peptidoglycan layer in the case of gram-positive bacteria and by the presence of lipopolysaccharides and porins on the outer membrane of the gram-negative bacteria [26]. The surface charge of liposomes can, e.g., be tuned by the introduction of lipids or amphiphiles with charged headgroups. 1,2-Dioleoyl-3-trimethylammonium-propane (DOTAP), 3β-[N-(N′,N′-dimethylaminoethane)-carbamoyl] cholesterol (DC-chol), or ethylphosphocholines are, e.g., commonly applied for the preparation of cationic liposomes [27–29]. As liposomes can be loaded with a variety of molecules, they have also been studied as carriers for antibiotics [30]. Therefore, also the electrostatic interaction between cationic liposomes and the respective bacteria is of major importance. Several studies confirm the successful interaction of cationic liposomes with bacteria like *Pseudomonas aeruginosa*, *Escherichia coli*, *Salmonella*, or *Staphylococcus aureus* [30–32]. However, these studies focus on the bactericidal action of cationic liposomes, whereas only few studies have been reported that exploit this property for the direct detection of bacteria. Petaccia et al., for example, introduced a fluorescent, surface potential-sensitive probe into the lipid bilayer which responds to the presence of some bacterial strains [33].

**Fig. 1:**
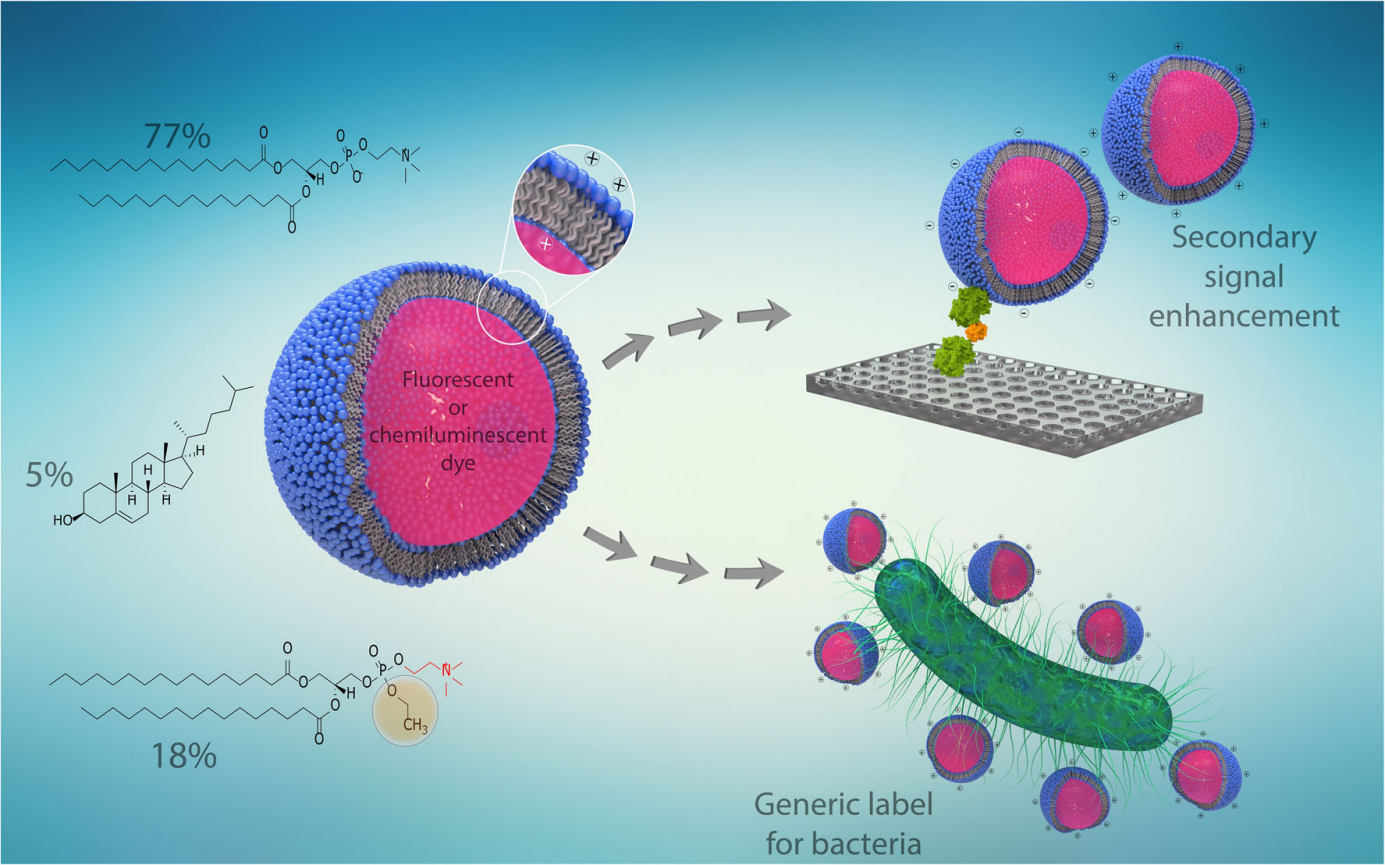
Optimized lipid composition for the preparation of cationic liposomes and investigated concepts

This study pursues a different approach which is solely based on the electrostatic interaction of cationic liposome surfaces, particles, and microorganisms to achieve an efficient labeling and signal amplification. Therefore, we aimed to develop highly stable, dye-loaded, cationic liposomes that can be applied as universal label in bioanalysis without the need for further functionalization with specific receptors. The synthesis of cationic liposomes described in this work can be regarded as a general protocol for enclosure of many different markers as encapsulants. Here, the cationic liposomes were either loaded with the fluorescent dye sulforhodamine B (SRB) or the chemiluminescent dye *m*-COOH-luminol. The functionality of the cationic liposomes as efficient label was proven via interaction with anionic liposomes and carboxylated, magnetic microparticles. Moreover, their ability to serve as secondary signal amplification tool was successfully shown. Finally, we took advantage of liposomes entrapping high concentrations of marker molecules and developed a labeling strategy for bacteria with *E. coli* as model analyte. We optimized liposome concentrations and studied various assay strategies including the use of fluorescent or chemiluminescent markers. It should be noted that these cationic liposomes will not provide selectivity as they are inherently generic by design. Instead, they are a unique opportunity for universal signal enhancement in bioassays (Fig. 1) with negatively charged analytes, where specificity and pre-selection is provided by capture molecules such as antibodies, aptamers, or receptor molecules.

## Materials and methods

### Materials

1,2-Dipalmitoyl-sn-glycero-3-phosphatidylcholine (DPPC), 1,2-dipalmitoyl-sn-glycero-3-ethylphosphocholine (EDPPC), 1,2-dipalmitoyl-sn-glycero-3-phospho-(1′-rac-glycerol) (DPPG), 1,2-dipalmitoyl-sn-glycero-3-phosphoethanolamine-*N*-(biotinyl), cholesterol, and the extrusion kit and membranes were purchased from Avanti Polar Lipids (avantilipids.com). The dialysis membrane spectra/por 4 with a MWCO of 12–14 kDa was purchased from spectrum labs (www.spectrumlabs.com). *E. coli* was purchased from the DSMZ (www.dsmz.de). 2-[4-(2-Hydroxyethyl)piperazin-1-yl] ethanesulfonic acid (HEPES), sodium azide, Sephadex G-50 medium, phosphate-buffered saline, sodium hydroxide, sodium chloride, sulforhodamine B monosodium salt (SRB), poly-l-lysine, hemin, and black microtiter plates from Nunc (Cat. No. 437111) were bought from Sigma-Aldrich (www.sigmaaldrich.com). White microtiter plates (Cat. No. 437796) were bought from Greiner Bio-One (www.gbo.com/de). Glycine and carboxylated, magnetic beads (PureProteome™ Carboxy FlexiBind 1 μm) were purchased from Merck (www.merckmillipore.com). *n*-Octyl-β-d-glucopyranoside (OG) and microtiter plates for the stability measurements were bought from Roth (Rotilabo-Mikrotestplatten, www.carlroth.com). For the investigations of the electrostatic binding, white streptavidin-coated microtiter plates C96 from Kaivogen (kaivogen.com) were used. *m*-COOH-luminol was synthesized and kindly provided by the group of Prof. Dr. Jacobi von Wangelin (University of Hamburg). Lysogeny broth (LB) was purchased from Alfa Aesar (www.alfa.com/de). Bacteriological agar, hydrogen peroxide, potassium hydrogen carbonate, sucrose, disodium hydrogen phosphate, sodium dihydrogen phosphate, methanol, chloroform, and all other chemicals were of analytical grade and purchased from VWR (de.vwr.com).

Millipore water (≥ 18.2 MΩ cm) was used for the preparations of all buffers and aqueous solutions needed for liposome preparation.

### Methods

#### Optimized synthesis of cationic liposomes by reverse phase evaporation

DPPC (17.3 mg), EDPPC (4.5 mg), and cholesterol (0.6 mg) were dissolved in chloroform (3 ml) and methanol (0.5 ml) in a 50-ml round bottom flask and sonicated at 60 °C for 1 min. Two milliliters of an aqueous solution containing either sulforhodamine B (SRB, 10 mM, dissolved in 210 mM NaCl, 0.02 M HEPES, pH 7.5) or *m*-COOH-luminol (25 mM, dissolved in 0.2 M HEPES, pH 8.5) was added and the mixture sonicated at 60 °C for 4 min. The organic solvent was removed by using a rotary evaporator at 60 °C and a pressure of 750 mbar for 40 min. Rotary evaporation is a critical step in liposome synthesis where it needs to make sure that the temperature is held above the phase transition temperature of all lipids (here 60 °C). The solution was vortexed, and another 2 ml of the aqueous solution was added. After vortexing, again the solution was rotated at 60 °C and 750 mbar for 20 min and then again at 60 °C and 400 mbar for 20 min. This procedure leads to the evaporation of the organic solvent and ensures that most of the aqueous solvent remains in the flask to contain the formed liposomes. The dispersion was being extruded through polycarbonate membranes (1 μm and 0.4 μm) at 60 °C by pushing the syringes back and forth 21 times for each membrane. Excess of the marker molecules was removed by size exclusion chromatography with a Sephadex G-50 column followed by dialysis for 24 h against HEPES-saline-sucrose (HSS) buffer (10 mM HEPES, 200 mM NaCl, 200 mM sucrose, 0.01% NaN_3_, pH 7.5, in the case of sulforhodamine B) or glycine-NaOH buffer (10 mM glycine, 200 mM NaCl, 114 mM sucrose, 0.01% NaN_3_, pH 8.6, in the case of *m*-COOH-luminol).

#### Optimized synthesis of anionic liposomes by reverse phase evaporation

According to the protocol reported by Edwards et al. [34], DPPC (15 mg), DPPG (7.5 mg), and cholesterol (10 mg) were dissolved in chloroform (3 ml) and methanol (0.5 ml) in a 50-ml round bottom flask and sonicated at 45 °C for 1 min. For the preparation of biotinylated liposomes, 1,2-dipalmitoyl-sn-glycero-3-phosphoethanolamine-*N*-(biotinyl) (2 mg) were added additionally. Next, 2 ml of encapsulant solution (either a sodium chloride solution (300 mM dissolved in 0.02 M HEPES) or a SRB solution (150 mM dissolved in 0.02 M HEPES)) was added and the mixture sonicated at 45 °C for 4 min. The organic solvent was removed by using a rotary evaporator at 45 °C and a pressure of 380 mbar for 40 min. The solution was being vortexed, and another 2 ml of the SRB solution was added. After vortexing again, the solution was rotated at 45 °C and 380 mbar for 20 min and then again at 45 °C and 280 mbar for 20 min. The solution was being extruded through polycarbonate membranes (1 μm and 0.4 μm) at 50 °C by pushing the syringes back and forth 21 times for each membrane. Excess of SRB was removed by size exclusion chromatography with a Sephadex G-50 medium column (24 × 12.5 cm) followed by dialysis for 24 h against HSS buffer.

#### Determination of size and zeta potential

Dynamic light scattering (DLS) and ζ-potential measurements were carried out on a Malvern Zetasizer Nano-ZS (www.malvern.com). For all measurements, the temperature was set to 25 °C. Semi-micro polymethyl methacrylate (PMMA) cuvettes (Brand) were used for size determinations, disposable folded capillary cells (Malvern) for the ζ-potential measurements. Before the measurements, the samples were diluted 1:100. As setting for liposomes, a n_D_^20^ of 1.34 and an Abs of 0.000 was selected and HSS buffer (n_D_^20^ = 1.342, viscosity = 1.1185 kg m^−1^ s^−1^, dielectric constant 78.5) was used as dispersant. An equilibration time of 60 s was applied before each measurement.

#### Determination of phospholipid concentration

The phospholipid concentration was determined by using a Spectroflame EOP inductively coupled plasma optical emission spectrometer (ICP-OES) from Spectro (www.spectro.com). Phosphorus was detected at either 177.495 nm or 213.618 nm with axial plasma observation. For this purpose, 20 μl of the liposome sample was diluted in 2980 μl of 0.5 M HNO_3_ (Suprapur® quality from Merck) and could then be used for the measurement. 0.5 M HNO_3_ and a solution of 100 μM of PO_4_^3−^ in 0.5 M HNO_3_ were used for calibration before each measurement.

#### Determination of the encapsulation efficiency

In order to determine the encapsulation efficiencies of SRB-encapsulating liposomes, the emission was read out with a FLUOstar OPTIMA microplate reader from BMG LABTECH (www.bmglabtech.com) at an excitation wavelength (λ_exc_) of 544 nm, an emission wavelength (λ_em_) of 575, and a gain of 1200 before and after lysing the liposomes with *n*-octyl-β-d-glucopyranoside (OG). Liposome dispersions were diluted 1:1000 either in HSS buffer or 30 mM OG and the concentration of SRB in the samples was determined via a calibration curve of different SRB standard solutions. The encapsulation efficiency in percent was calculated using Eq. 1.

1 $$ \mathrm{Encapsulation}\ \mathrm{Efficiency}=\frac{c\left(\mathrm{after}\ \mathrm{lysis}\right)-c\left(\mathrm{before}\ \mathrm{lysis}\right)}{\mathrm{total}\ \mathrm{amount}\ \mathrm{of}\ \mathrm{SRB}}\cdotp 100\% $$

with *c* (after lysis) = concentration of dye in solution after liposome lysis, *c* (before lysis) = concentration of dye in solution before liposome lysis, total amount of SRB = concentration of dye solution that was applied for entrapment.

#### Liposome stability regarding dye leakage

The leakage of SRB from the liposomes was monitored via fluorescence analysis. Liposome samples were diluted 1:1000 once in HSS buffer and once in an OG solution (30 mM in HSS). For fluorescence analysis, the liposome dilutions were added to a 96-well plate (200 μl/well, 3 replicates). The emission was read out with a FLUOstar OPTIMA microplate reader (BMG Labtech) at an excitation wavelength (λ_exc_) of 544 nm, an emission wavelength (λ_em_) of 575 nm, and a gain of 1200. Liposome lysis in percent was calculated using Eq. 2.

2 $$ \mathrm{Lysis}\ \left[\%\right]=\frac{I_{\mathrm{F}}-{I}_0}{I_{100\%}-{I}_0}\cdotp 100\% $$

with *I*_F_ = fluorescence intensity in liposome sample, *I*_0_ = fluorescence of reference solution (only liposomes), *I*_100%_ = fluorescence intensity after complete lysis of liposomes with OG.

#### Binding to streptavidin-coated microtiter plates

Binding to anionic liposomes was investigated via fluorescence measurements on streptavidin-coated microtiter plates. First, a dispersion of sodium chloride encapsulating, anionic, biotinylated liposomes (100 μl/well, 10 μM in 1×HSS buffer) was added to the wells of the microtiter plate. The plate was incubated at room temperature for 1 h in the dark. After washing with 1×HSS buffer (200 μl) three times, different concentrations of cationic SRB-encapsulating liposome solutions were added (0–100 μM in 1×HSS buffer, 100 μl/well) and incubated again for 1 h. After washing again with 1×HSS buffer (200 μl/well), 100 μl of a 30 mM *n*-octyl-β-d-glucopyranoside (OG) solution was added to the wells. The fluorescence intensity was read out with a FLUOstar OPTIMA microplate reader (λ_exc_ = 544, λ_em_ = 575 nm, gain 1200). A negative control experiment was conducted by adding the cationic liposomes directly to the streptavidin-coated plate. Three replicates of each concentration of cationic liposomes were made for both experiments.

#### Magnetic bead studies

Phosphate buffer (10 mM, pH 6) was used to prepare dispersions containing cationic liposomes between concentrations of 0 and 200 μM in the wells of a 96-well plate (100 μl/well). Then a 1:10 dilution of carboxyl magnetic beads was added (10 μl/well, 10 mg ml^−1^ stock solution). After incubation at 23 °C and shaking at 500 rpm for 30 min, the bead-liposome aggregates were separated using a magnetic plate. The supernatant was removed, and the residual aggregates washed twice with HSS buffer (200 μl/well). The fluorescence signal was read out with a FLUOstar OPTIMA microplate reader (BMG Labtech) at wavelengths of λ_exc_ = 544 nm and λ_em_ = 575 nm and a gain of 1500 before (in 100 μl HSS, background) and after lysis with 30 mM *n*-octyl-β-d-glucopyranoside (OG, 100 μl). Three individual measurements of each dilution of cationic liposomes (0–200 μM) were made. For analysis, the background fluorescence was subtracted from the intensity after lysis.

#### Preparation of *E. coli* cultures

*E. coli* was cultivated in LB medium (10 ml) over night at 37 °C under continuous shaking. One milliliter of the bacteria solution was then centrifuged for 5 min at 1500 rcf and the pellet resuspended in PBS buffer. For colony counting, this stock solution was diluted 1:10^6^ in LB medium and 50 μl of this solution spread onto an Agar plate and incubated overnight at 37 °C. This was done for 3 different plates and the grown colonies were counted the next morning.

#### Microtiter plate coating with poly-l-lysine

Poly-l-lysine (200 μl/well, 50 μg ml^−1^ in PBS) was added to the wells of a white (for chemiluminescence analysis) or black MaxiSorp microtiter plate (for fluorescence analysis) and incubated overnight at 4 °C. Before using the plate, the poly-l-lysine solution was removed, and the wells washed twice with PBS buffer.

#### Bacteria detection assay

#### Bacteria assay on poly-l-lysine-coated microtiter plates

The *E. coli* stock solution was transferred into PBS buffer and diluted to concentrations between 0 and 10^8^ colony forming units (cfu) ml^−1^. The dispersions were then incubated in the wells of a poly-l-lysine-coated microtiter plate at room temperature for 1 h. After washing twice with PBS buffer, cationic liposomes (50 μM in PBS, 100 μl/well) were added and incubated for 1 h at room temperature. The wells were then washed twice with PBS buffer (200 μl/well). For fluorescence analysis, PBS buffer (100 μl/well) was added and the fluorescence read out with a FLUOstar OPTIMA microplate reader (BMG Labtech) (λ_exc_ = 544 nm and λ_em_ = 575 nm, gain 1200) before (background) and after lysis of the liposomes with *n*-octyl-β-d-glucopyranoside solution (OG, 300 mM in PBS, 10 μl/well). Three individual measurements of each *E. coli* dilution were made. For analysis, the background fluorescence was subtracted from the intensity after lysis.

#### Bacteria assay with centrifugation

##### Fluorescence analysis

The *E. coli* stock solution was transferred into PBS buffer. Six different dilutions (1 ml each; 1:10, 1:100, 1:1000, 1:10^4^, 1:10^5^, and 1:10^6^) of the *E. coli* solutions were made in phosphate buffer (10 mM, pH 6) and cationic liposomes were added to them (50 μM in sample). The mixture was centrifuged at 1500 rcf for 10 min. After removal of the supernatant, the pellet was redispersed in 1 ml *n*-octyl-β-d-glucopyranoside solution (30 mM). Fluorescence spectra of SRB-encapsulating liposomes were recorded between 560 and 654 nm with λ_exc_ = 544 nm and a detector voltage of 550 V on an Aminco-Bowman Series 2 (AB2) instrument (Thermo Electron Corporation). Three individual measurements of each *E. coli* dilution were made.

##### Chemiluminescence analysis

The *E. coli* stock solution was transferred into PBS buffer and diluted to concentrations between 0 and 10^8^ cfu ml^−1^. The dispersions were then incubated with cationic liposomes (50 μM) for 4 h at room temperature. Then, the mixture was centrifuged for 30 min at 1500 rcf, the supernatant removed, and the residuum resuspended in PBS buffer (500 μl). This step was repeated twice; after the last centrifugation, the residuum was resuspended in carbonate buffer (0.1 M, 100 mM NaCl, pH 10.5, 500 μl) containing 30 mM OG. For the measurement, the mixture was added to the wells of a white microtiter plate (100 μl/well). Hemin (10 μM, 1 μl/well) was added as catalyst for chemiluminescence analysis. H_2_O_2_ (2 mM, 2 μl/well) was added right before the measurement. Three individual measurements of each *E. coli* dilution were made.

A BioTek microplate reader was used for all chemiluminescence measurements with *m*-COOH-luminol-containing liposomes and the read height was adjusted to 6 mm. The gain was adjusted for each experiment (as indicated in the respective figures) and was always between 60 and 80.

## Results and discussion

The cationic liposomes used in this work were synthesized by reverse phase evaporation. The membrane of the developed positively charged vesicles is mainly composed of DPPC, which is a common zwitterionic phospholipid applied in liposome formulations. The addition of cholesterol helps to prevent leakage of entrapped molecules as this lipid is able to influence the membrane fluidity [35–37]. EDPPC is a synthetic cationic lipid that derives from its natural precursor DPPC and belongs to the class of phosphatidylcholine triesters. Its phase transition temperature is at 41 °C similar to that of DPPC [38] and is therefore ideally suited to give the vesicles an overall positive surface charge.

Our aim was to create marker molecule (such as sulforhodamine B, SRB, or luminol) encapsulating cationic liposomes that provide a similar excellent long-term colloidal stability, vesicle diameter, and minimized dye leakage like anionic liposomes developed by Edwards et al. [7]. Therefore, variations in the lipid composition were studied, specifically of the EDPPC and cholesterol content. The liposomes were characterized by DLS, ζ-potential, phospholipid concentration, and encapsulation efficiency. A content of 10 mol% EDPPC was found to be insufficient to create positively charged liposomes that do not aggregate immediately. When raised to 18 mol% and combined with a cholesterol content of 5% and 77 mol% DPPC, highly stable cationic liposomes were generated with average diameters of 300 nm and a zeta potential of + 18 mV (Electronic Supplementary Material (ESM) Table S1). At higher concentrations of cholesterol, even at only 10 mol%, the formation of positively charged liposomes was inhibited. Moreover, it was found that the addition of sodium chloride to the encapsulant solution of the SRB liposomes resulted in a decrease in diameter below 200 nm (ESM Table S1, Fig. S1) and a significant increase in the phospholipid concentration as well as in the encapsulation efficiency from 3% to almost 6% as presented in ESM Table S1. It has been found previously that NaCl assists in the colloidal stabilization of anionic liposomes. It is assumed that the additional electrolyte presence assists in stabilizing the lipid-water interface during the evaporation process. The superior performance of the cationic liposomes entrapping a mixture of SRB and sodium chloride can also be observed regarding their colloidal long-term stability. Here, no change in the vesicle diameter was observed so far, while cationic vesicles that do not entrap any sodium chloride tend to agglomerate over time (ESM Table S2). The optimized lipid composition and the investigated concepts are shown in Fig. 1.

**Fig. 2:**
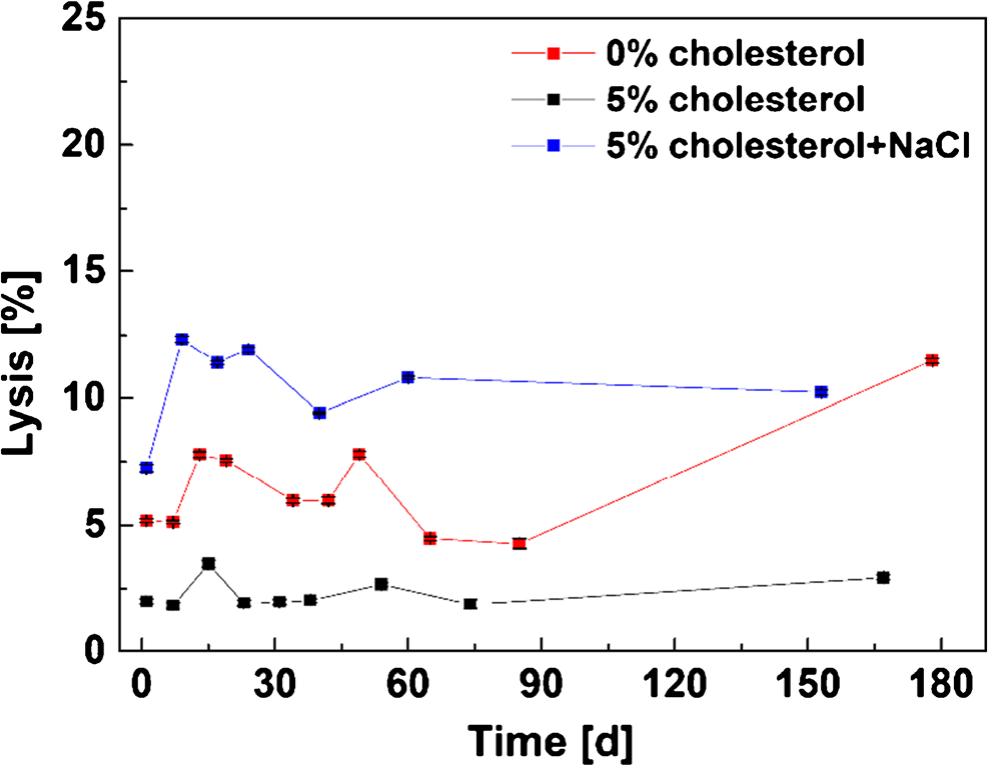
Dye leakage of SRB-encapsulating cationic liposomes determined via fluorescence before and after lysis of the liposomes with 30 mM OG. All liposome preparations contain 10 mM SRB. The 3rd variation (5% cholesterol+NaCl) entraps additionally 210 mM NaCl. Fluorescence intensities were read out on a FLUOstar OPTIMA microplate reader at *λ*_exc_ = 544 nm and *λ*_em_ = 575 nm and a gain of 1200, 3 replicates (error bars are provided, but are in some instances smaller than the chosen size of the symbols)

The long-term stability of the liposomal membrane was investigated using a dye leakage assay. Therefore, the release of the entrapped SRB was monitored over a period of 5 months and the liposome lysis was calculated using Eq. 2. Figure 2 shows that positively charged liposomes overall are stable for up to 90 days with no significant change in observed lysis. Not surprisingly, liposomes not containing cholesterol (red curve) show degrees of lysis at 180 days with a doubling of the observed fluorescence generated by free dye. It should be noted that the difference in observed fluorescence signal between the three types of liposomes studied (i.e., values of 2%, 5%, and 7%) is not an indication of free dye, as additional purification steps were not able to reduce the higher values. We therefore assume that SRB molecules are ionically associated with the outer membrane of the cationic liposomes.

Since these optimized vesicles show similar properties as standard dye encapsulating anionic liposomes that are often applied in bioanalysis [7, 18, 20], we concluded that they should also be highly suitable for the development of bioassays. Specifically, assay types in which analyte specificity is already afforded through other binding events such as through antibodies, aptamers, or receptor molecules will be highly applicable for the cationic liposomes as universal label. We hence studied a variety of such assay types (Fig. 1).

**Fig. 3:**
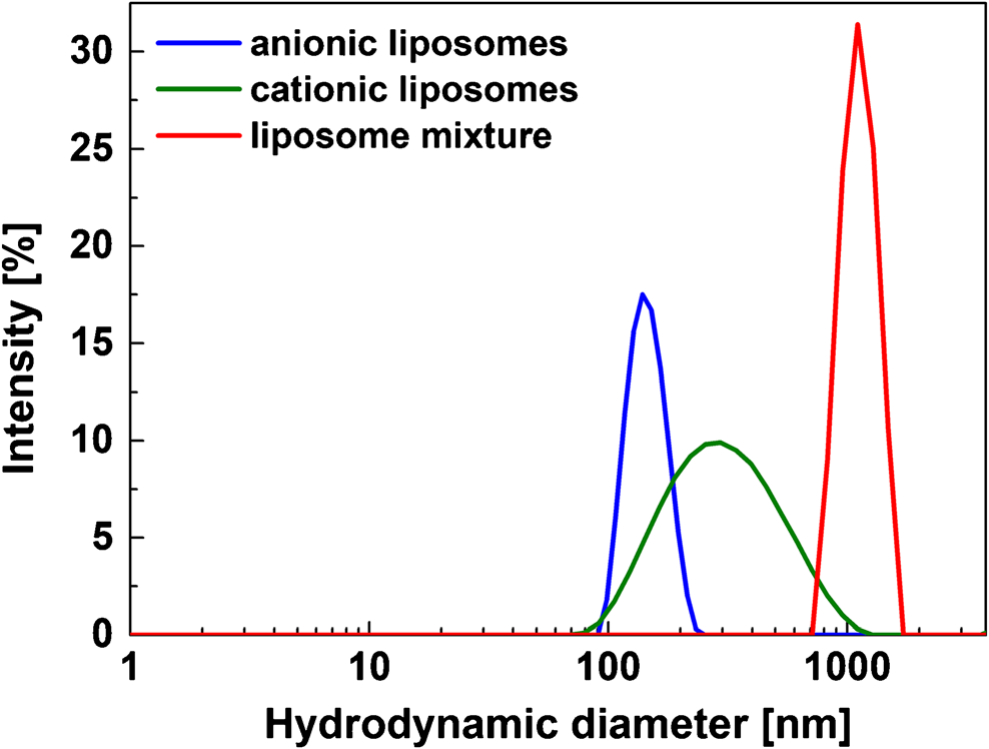
Dynamic light scattering analysis of the diameter of the cationic SRB-encapsulating liposomes without cholesterol (phospholipid concentration 1.10 mM), anionic SRB-encapsulating liposomes with (phospholipid concentration 1.30 mM) and a mixture (cationic:anionic 3:1) of the two of them in HSS buffer (10 mM HEPES, 200 mM NaCl, 200 mM sucrose, 0.01% NaN_3_, pH 7.5) determined by DLS

**Fig. 4:**
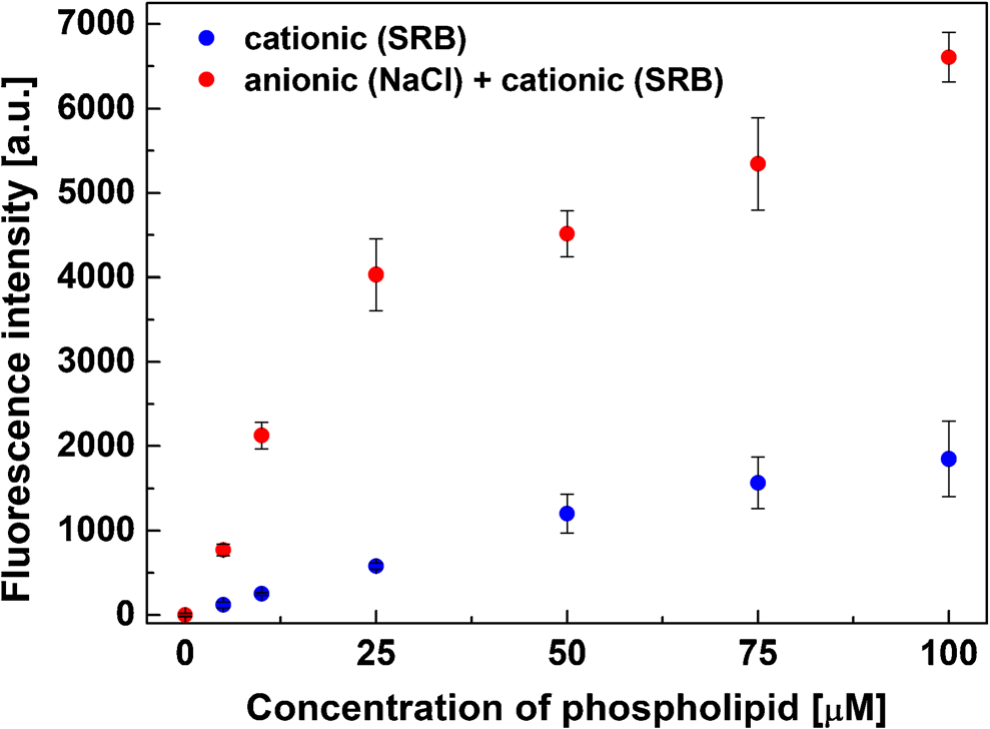
The electrostatic binding of cationic SRB-encapsulating liposomes to anionic, biotinylated, colorless liposomes was investigated. The colorless anionic liposomes were bound to a streptavidin-coated microtiter plate. Electrostatic binding of the SRB-encapsulating cationic liposomes was determined by fluorescence measurements before and after lysing the liposomes. Fluorescence intensities were read out on a FLUOstar OPTIMA microplate reader at λ_exc_ = 544 nm and λ_em_ = 575 nm and a gain of 1200, 3 replicates

For example, cationic liposomes could be used as secondary additional label on anionic liposomes, if further signal improvement is necessary. Thus, the electrostatic interaction itself was investigated by binding the vesicles to negatively charged liposomes of similar size and was successfully demonstrated using DLS and fluorescence measurements (Figs. 3 and 4).

The DLS spectra (Fig. 3) show only one peak in the presence of anionic (180 nm) or cationic liposomes (300 nm), respectively. However, electrostatic binding between the differently charged liposomes leads to the expected bigger aggregates. An excess of cationic liposomes was chosen to yield aggregates with anionic liposomes in the center that are surrounded by cationic liposomes, which was supported by DLS and zeta potential analyses. After mixing of the two vesicle dispersions, the DLS peaks representing the liposomes are combined to a single peak at higher values with an average diameter of 1105 nm and a PdI of 0.041 (Fig. 3), which indicates the formation of vesicle agglomerates averaging a total of 4 liposomes per aggregate. In addition, no individual liposomes can be found in the intensity-weighted distribution anymore (Fig. 3). The same could be concluded from the number-weighted distribution (ESM Fig. S2). Another indication for the formation of defined aggregates where positively charged liposomes are “surrounding” the anionic liposomes can be found by looking at the zeta potential which shows a positive value of + 8 mV after mixing of the two types of liposomes. Consequently, the attraction between the two counter charged vesicles is high enough to cause aggregation, which supports the strategy to use cationic liposomes as secondary label in liposome-based bioassays.

This applicability was then tested in a biotin assay using a microtiter plate coated with streptavidin. Biotinylated, colorless, anionic liposomes were bound to this surface, free liposomes removed via washing, and the surface then incubated with varying concentrations (0 to 100 μM) of cationic SRB-encapsulating liposomes. Here, the anionic liposomes entrapped no dye but only 300 mM NaCl and hence produced no fluorescent signal, so that any fluorescence is directly attributed to the secondary, cationic liposomes. As expected, strongly increasing fluorescent signals are obtained with increasing cationic liposome concentrations (Fig. 4). A negative control experiment was conducted by adding the cationic liposomes directly to the streptavidin-coated plate. Here, a small increase in the signals of the negative controls is likely caused by cationic liposomes adsorbing non-specifically to the surface. However, even without any further surface blocking, by simply adding 10 μM of SRB-encapsulating cationic liposomes, a signal to noise ratio of 8.5 is obtained which would accordingly enhance any liposome-based bioassay significantly.

In a second assay, liposome binding to other negatively charged particles was investigated to proof the applicability of the cationic vesicles as universal label for negatively charged particles. Here, magnetic beads were chosen as they afford simple separation strategies. Also, of special interest here is the larger size of the magnetic beads (1 μm in diameter), which is similar to bacterial cells, and lysis of cationic liposomes through rupture and agitation due to the beads’ movement within the magnetic field could be studied.

**Fig. 5:**
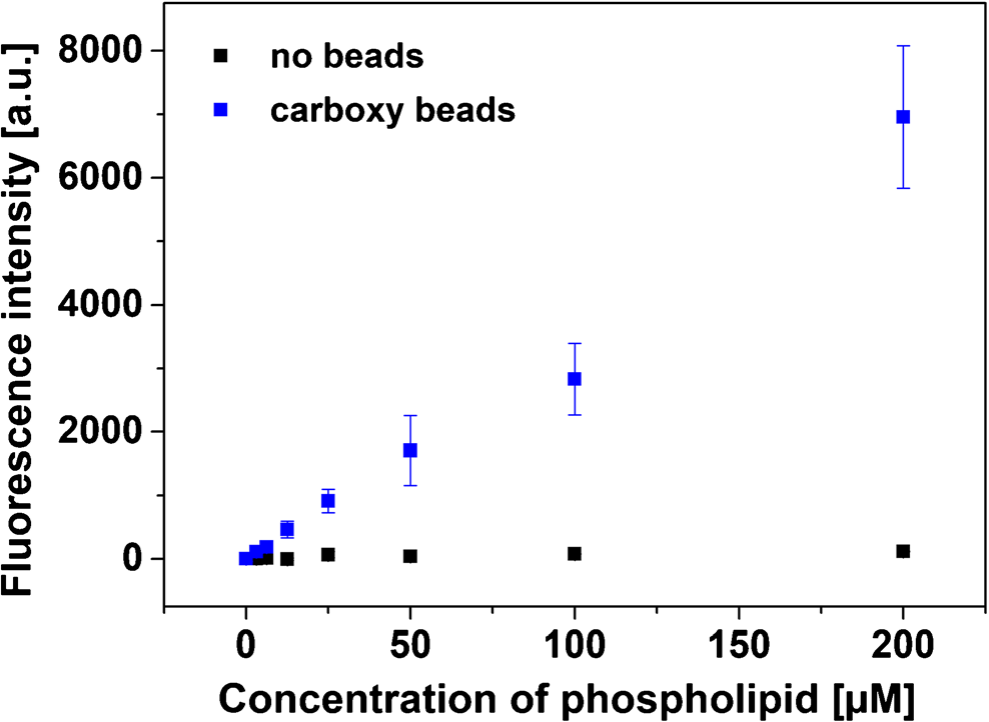
Different concentrations of cationic SRB-encapsulating liposomes were incubated with magnetic carboxyl beads. Electrostatic interaction leads to strong binding and easily quantifiable signals. Fluorescence intensities were read out after a washing step on a FLUOstar OPTIMA microplate reader at *λ*_exc_ = 544 nm and *λ*_em_ = 575 nm and a gain of 1500, 3 replicates (error bars are provided, but are in some instances smaller than the chosen size of the symbols)

For this purpose, different concentrations of cationic liposomes entrapping the fluorescent dye sulforhodamine B were mixed with the carboxyl group bearing magnetic beads. Unbound material was separated magnetically after an incubation of 30 min at room temperature. As negative control, the same was done for liposomes without the addition of magnetic beads. Then, the fluorescence intensity of the bound liposomes was analyzed after lysis of the vesicles (Fig. 5). As expected, no signal was obtained in the case of the negative control experiment (i.e., no non-specific binding of cationic liposomes) but a significant increase in the fluorescence intensity was observed with an increasing concentration of initially supplied liposomes. Furthermore, no lysis (i.e., free SRB dye) was detected after the incubation and magnetic separation suggesting a very high liposome assay stability. Thus, cationic liposomes can also be integrated in magnetic separation assays and serve as universal labeling agent.

In a final example, the applicability of the cationic liposomes to interact with an actual biological analyte is shown. Therefore, the vesicles were used for the ionic interaction-based labeling of gram-negative bacterial cells. To prove the negative surface charge of the *E. coli* cells, ζ-potential measurements were conducted in phosphate buffer for a pH range from 4 to 9. Cationic liposomes were investigated in the same range to find the optimum pH for all further measurements (ESM Fig. S3). The ζ-potential values for the *E. coli* cells are always in the range from − 40 to − 50 mV with no significant influence of the pH. In contrast, the ζ-potential of the cationic liposomes decreases with increasing pH from + 55 to + 20 mV. However, as too acidic conditions may compromise the stability of the liposomes and the *E. coli* cells during incubation, pH values between 6 and 7 were used for all further experiments. Under these conditions, the expected ionic interaction between the two species could easily be observed.

**Fig. 6:**
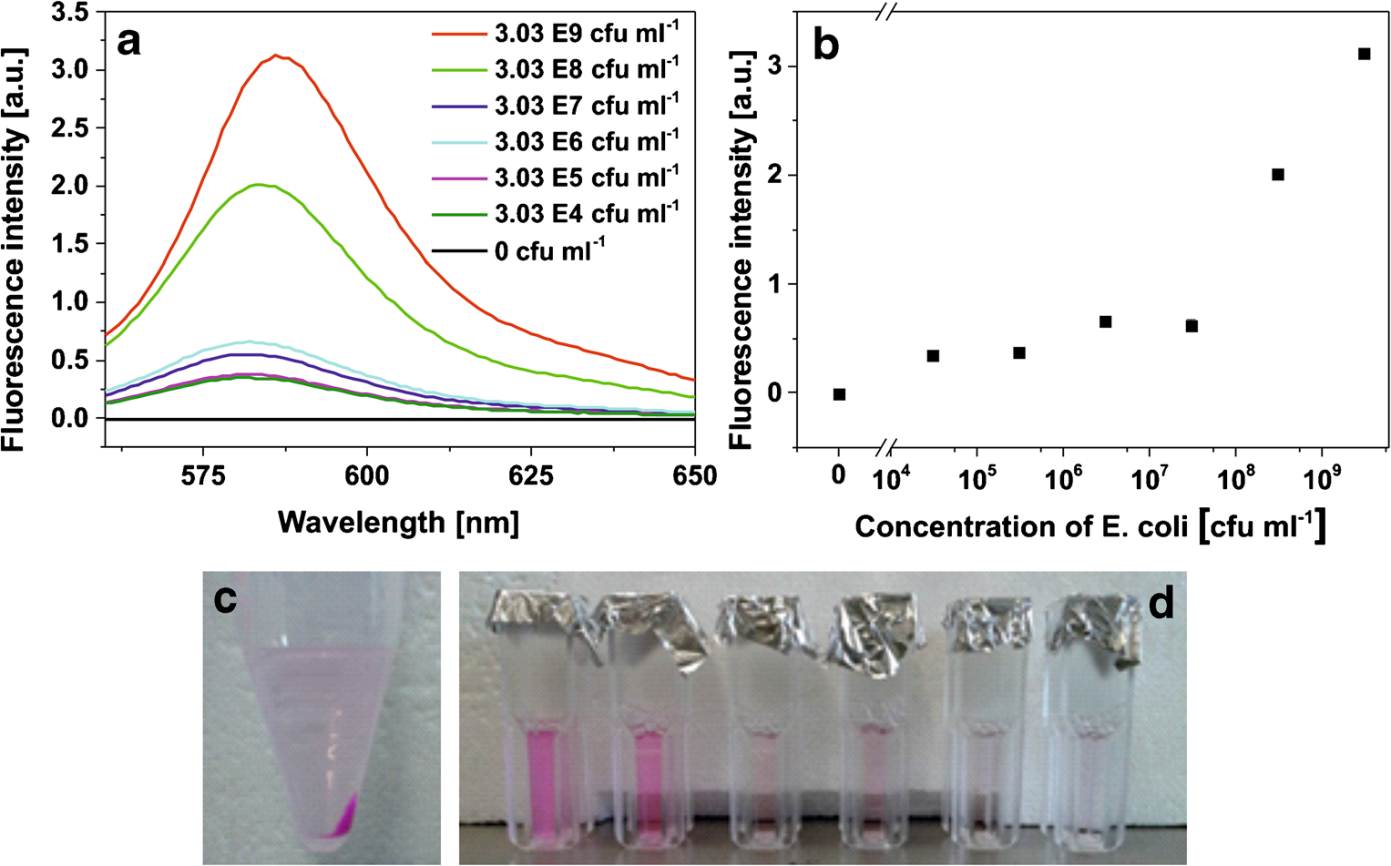
**a** Fluorescence spectra and **b** the corresponding intensities at the peak maximum of lysed liposomes after incubation with *E. coli* cells and separation via centrifugation. Fluorescence spectra were recorded between 560 and 650 nm using an Aminco-Bowman 2 spectrofluorometer at *λ*_exc_ = 544 nm and *λ*_em_ = 575 nm and a detector voltage of 550 V, 3 replicates (error bars are provided, but are in some instances smaller than the chosen size of the symbols). **c** Formation of pink pellet after centrifugation. **d** Liposome-*E. coli* agglomerates after centrifugation and redispersion in 30 mM OG for *E. coli* concentrations between 3E09 and 3E04 cfu ml^−1^ (from left to right)

Specifically, when different concentrations of *E. coli* cells were mixed with cationic SRB-encapsulating liposomes in a cuvette, pink agglomerates were observed to settle to the bottom of the cuvette after an incubation of 2 h for a bacteria concentration of 10^8^ cfu ml^−1^ (ESM Fig. S4). For lower concentrations (10^5^ cfu ml^−1^), this agglomeration was not visible by bare eye anymore. Hence, to separate the vesicle-bacteria agglomerates from unbound liposomes, centrifugation was applied resulting in expected pink pellets (Fig. 6c). When washed and then lysed to release entrapped SRB, as few as 10^4^ cfu ml^−1^*E. coli* cells could easily be discerned from the background signal (Fig. 6d). However, it was not possible to develop a quantitative and reliable assay using this simple centrifugation-based separation step as the pellets generated by low cell concentrations were fluffy and only weakly associated, so that washing and repelleting leads to high variations in the obtained signals. This is obvious when comparing signals generated by 10^4^ cfu ml^−1^ and 10^5^ cfu ml^−1^, which are not discernible (Fig. 6 a and b).

**Fig. 7:**
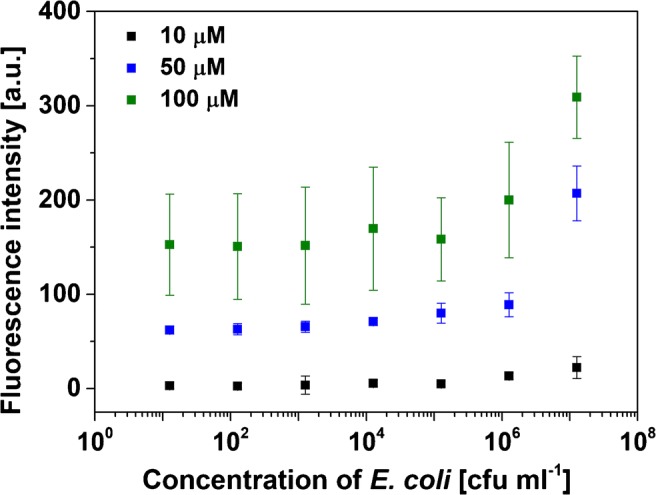
Labeling of *E. coli* cells immobilized via poly-l-lysine in microtiter plates. Different concentrations of cationic SRB-encapsulating liposomes and bacterial cells were studied. Fluorescence was read out on a FLUOstar OPTIMA microplate reader at *λ*_exc_ = 544 nm and *λ*_em_ = 575 nm and a gain of 1200, 3 replicates (error bars are provided, but are in some instances smaller than the chosen size of the symbols)

In order to optimize the liposome labeling strategies and develop it to be reliable and quantifiable, a better separation strategy (other than centrifugation) was sought. It was found that *E. coli* cells could easily be immobilized in microtiter plates by binding to the positively charged polymer poly-l-lysine. At the same time, poly-l-Lysine prevents the cationic liposomes from binding to the plate when no bacteria are present. The cells were then first bound to the poly-l-lysine-coated microtiter plates and subsequently incubated with the SRB-encapsulating cationic liposomes. Buffer optimization studies showed that binding of the liposomes to *E. coli* cells was best in PBS (ESM Fig. S5) while incubation in LB medium, sucrose-containing buffer or water led to unwanted interferences with medium and buffer components or the disruption of the vesicles, respectively. Moreover, the electrostatic interaction seems to be reduced at higher salt concentrations (200 mM NaCl). For the optimization of liposome labeling, it was then found that too low concentrations of phospholipid (10 μM) did not provide a detectable signal, and too high concentrations (100 μM) lead to unreliable results (Fig. 7). Interestingly, higher phospholipid concentrations also had a bactericidal effect (Table 1). Thus, 50 μM phospholipid concentrations was chosen for all further studies.

**Table 1:** Colonies of *E. coli* grown overnight after a 1-h incubation with cationic SRB-encapsulating liposomes

*c* (Cationic liposomes) (μM)	Counted colonies
0	121 ± 18
10	115 ± 19
50	108 ± 20
100	91 ± 14
200	96 ± 1

Moreover, the influence of the cationic surface charge and fluidity of the liposomal membrane was studied by increasing the EDPPC or cholesterol concentration. We hypothesized that a higher charge might be able to enhance the interaction with the bacteria and thus result in lower limits of detection. ζ-Potential measurements confirmed the successful incorporation of an increased amount of EDPPC via an increase of the surface potential from + 15 mV to + 25 mV and to + 32 mV in the case of a 50:50 mixture of EDPPC and cholesterol (ESM Fig. S6). However, these variations did not render the liposome better with respect to ionic interaction with the bacterial cells as shown for the liposome-bacteria centrifugation assay (ESM Fig. S7). Instead, the higher positive surface charge may have led to an increased colloidal stability of the vesicles that rather prevents agglomeration with other particles.

Finally, the entrapped marker molecules were altered changing the signaling strategy from fluorescence to the more sensitive chemiluminescence. Specifically, *m*-COOH-luminol was used as encapsulant adapting previously described chemiluminescent anionic liposomes [10]. This resulted in *m*-COOH-luminol-containing cationic liposomes with a diameter of 142 nm, a low PdI of only 0.13, and a ζ-potential of + 15 mV (ESM Fig. S8a). The cationic features compared nicely with those of the SRB-encapsulating liposomes (ESM Figs. S1 and S9) and the *m*-COOH-luminol entrapment yield (ESM Fig. S8b) well to those of the previous anionic ones [10].

**Fig. 8:**
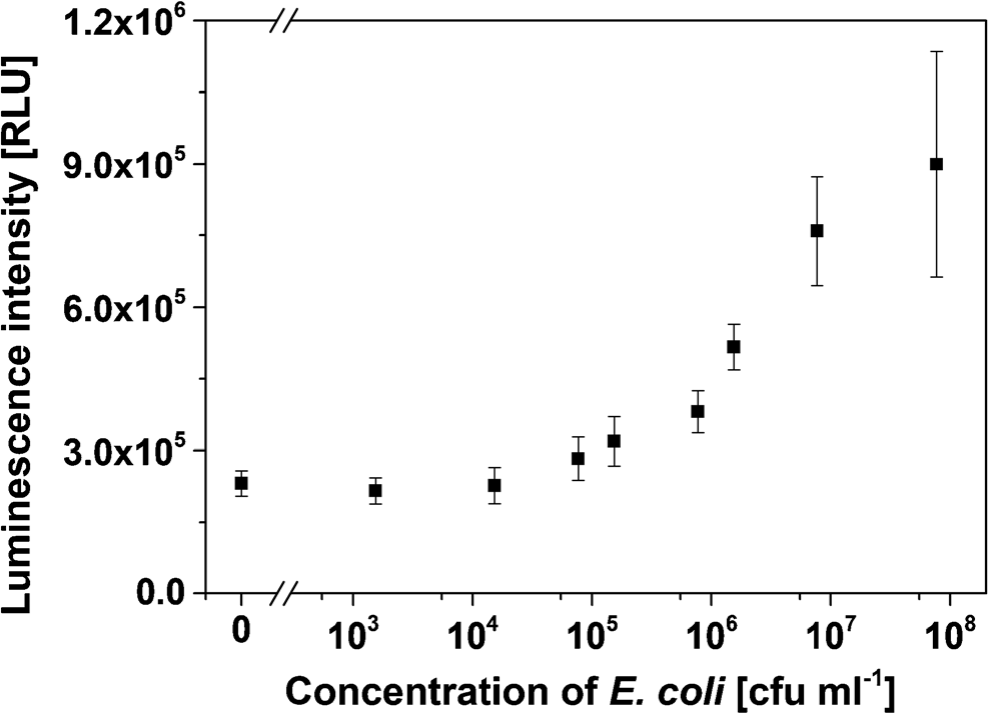
Detection of *E. coli* cells with cationic *m*-COOH-luminol-containing liposomes. Liposome-cell complexes were separated from free liposomes via simple centrifugation. Chemiluminescence measurements were conducted in 30 mM OG in carbonate buffer (0.1 M, pH 10.5) on a BioTek microplate reader with hemin and H_2_O_2_ as co-reagents, a read height of 6 mm, and a gain of 80, 4 replicates

With the combination of all of these optimization strategies, we could prove that highly reliable and quantitative interactions of cationic liposomes with bacterial cells can indeed be accomplished. Specifically, when using these *m*-COOH-luminol-containing liposomes as universal ionic label for *E. coli* with centrifugation as separation strategy, a highly reliable bioassay was obtained (Fig. 8). This demonstrates that the low reliability observed when using SRB-encapsulating liposomes as described above can be overcome with a more sensitive detection strategy such as chemiluminescence. Based on all of these findings, specific bioassays can be developed in the future in which antibodies would be immobilized either on a microtiter plate or preferably on magnetic beads. Here, liposomes could then easily serve as universal and powerful signal amplification label.

## Conclusions

This work demonstrates the development of highly stable, positively charged SRB or *m*-COOH-luminol-encapsulating liposomes and their ability to be applied as label and signal amplification tool in bioanalytical systems based on electrostatic interactions. The applicability as secondary signal amplification tools in combination with anionic liposomes was proven via dynamic light scattering, which shows the agglomeration of the oppositely charged vesicles. In addition, fluorescence measurements on streptavidin-coated microtiter plates using biotinylated anionic liposomes and SRB-loaded cationic liposomes confirmed the DLS results. Moreover, the applicability of these cationic liposomes as label for bigger particles or microorganisms has been demonstrated via magnetic beads. Finally, it was possible to transfer these findings to a simple labeling strategy for *E. coli* cells, where the interaction between cationic liposomes and the bacteria was successfully demonstrated by agglomeration visible by bare eye, fluorescence, and chemiluminescence analysis.

These findings show that cationic liposomes can become a powerful tool in the field of bioanalysis. The simple labeling via electrostatic interaction is independent of additional recognition elements like DNA or antibodies, which reduces preparation costs and time. The vesicles can therefore be applied as universal label not only for bacteria but also for other negatively charged analytes as, for example, RNA or DNA molecules. Also, multimodal liposomes can be envisioned that simultaneously quantify and destroy bacterial cells via the loading of an antimicrobial agent into the lipid vesicles. Moreover, interactions between oppositely charged vesicles could be exploited for fusion-based bioassays. Finally, the synthesis for cationic liposomes described in this work may be used for many different marker molecules. By varying the type of encapsulant, the concept of electrostatically enhanced, liposome-based assays could find its applications in fluorescence, electrochemical, and electrochemiluminescence detection as well as for surface plasmon resonance and mass-sensitive detection techniques.

## Electronic supplementary material

ESM1 (PDF 48 s8 kb)
