# Supplementary material for: Cationic liposomes for generic signal amplification strategies in bioassays
Source: Anal Bioanal Chem. 2020 Apr 6;412(14):3383–93. doi: 10.1007/s00216-020-02612-w (PMC7214507; doi:10.1007/s00216-020-02612-w)
Supplement: Supplementary file 1 — (PDF 48 s8 kb) [file 216_2020_2612_MOESM1_ESM.pdf]

Analytical and Bioanalytical Chemistry

Electronic Supplementary Material

Cationic liposomes for generic signal amplification strategies in bioassays

Carola Hofmann, Barbara Kaiser, Susanne Maerkl, Axel Duerkop, Antje J. Baeumner

1. Results

Table S1 Comparison of three preparations of cationic liposomes with different lipid compositions

	**0% cholesterol, 18% EDPPC**	**5% cholesterol, 18% EDPPC**	**5% cholesterol, 18% EDPPC +NaCl**
**Diameter [nm]**	294 ± 7	311 ± 2	187 ± 3
**Zeta potential [mV]**	+20 ± 1.1	+18 ± 0.5	+16 ± 0.8
**Phospholipid concentration [mM]**	1.1 ± 0.01	1.1 ± 0.01	6.3 ± 0.03
**Lysis [%] after 165 days**	11 ± 0.09	3 ± 0.1	10 ± 0.1
**Encapsulation efficiency [%]**	3.5 ± 0.1	2.7 ± 0.09	5.7 ± 0.1

As stated in the main manuscript, the vesicle stability was also investigated by observing changes in the diameter of the three different types of liposomes as shown in Table S2. Cationic liposomes containing no sodium chloride in their interior tend to agglomerate over time. This may be due to the fact that the magnitude of their zeta potential is lower as in case of our standard anionic liposomes, which results in an increase in diameter over time due to agglomeration. The addition of cholesterol does not only prevent the leakage of SRB molecules through the membrane but also seems to endure the colloidal stability as here agglomeration can only be observed after six months. The higher salt concentration in the liposomes with NaCl seems to be able to prevent the agglomeration best. Here, no increase in diameter could be observed so far. Long-term stability for anionic liposomes has been shown to be more than one year [1] which is also expected for the optimized cationic NaCl-containing liposomes based on the current performance.

Table S2 Hydrodynamic diameters of anionic and cationic liposome dispersions determined right after the preparation and after 3 and 6 months by DLS, 3 replicates

Preparation	Diameter [nm]		
	Day 1	3 months	6 months
**Cationic without cholesterol**	294 ± 7	848 ± 8	735 ± 40
**Cationic with 5% cholesterol**	311 ± 2	444 ± 18	736 ± 6
**Cationic with 5% + NaCl**	187 ± 3	187 ± 7	186 ± 4

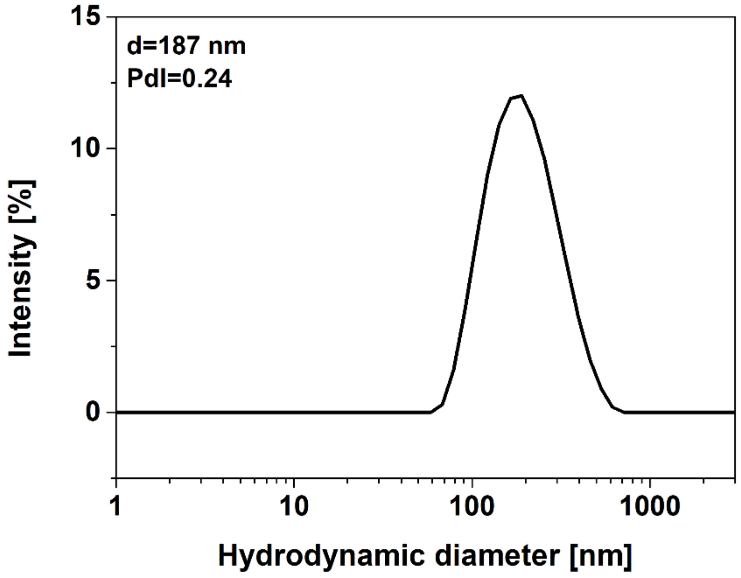

Fig. S1 Hydrodynamic diameter of cationic SRB-encapsulating liposomes in HSS buffer determined by DLS

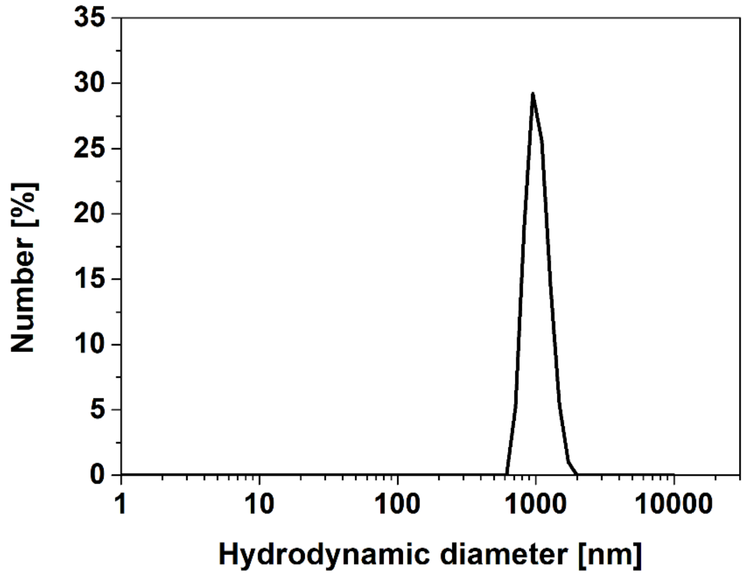

Fig. S2 Number-weighted distribution of the hydrodynamic diameter of the mixture of cationic and anionic liposomes in HSS buffer determined by DLS

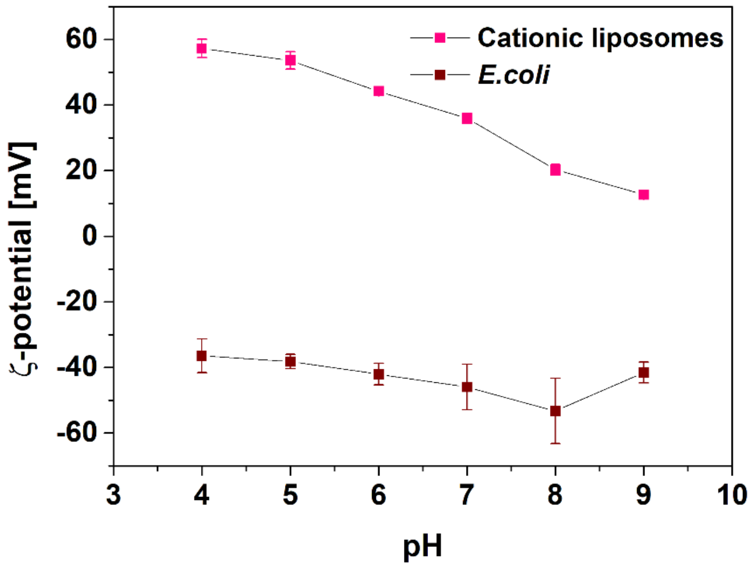

Fig. S3 ζ-potentials of E. coli cells and cationic liposomes dispersed in phosphate buffer in a pH range of 4-9, 4 replicates

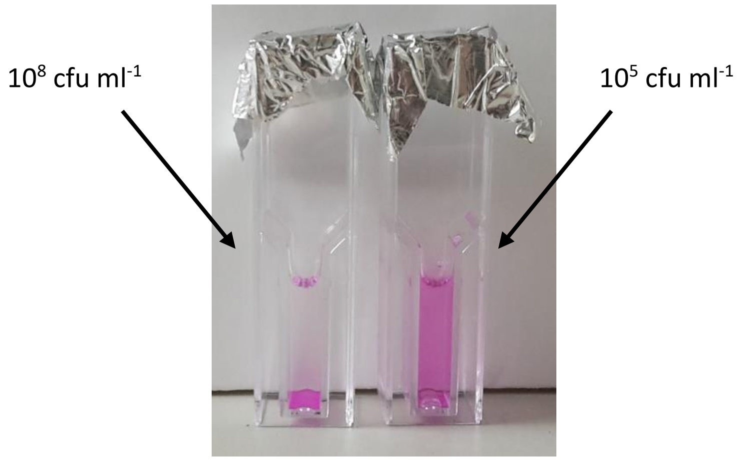

Fig. S4 Mixtures of cationic liposomes and different concentrations of E. coli after a 2 h incubation at room temperature

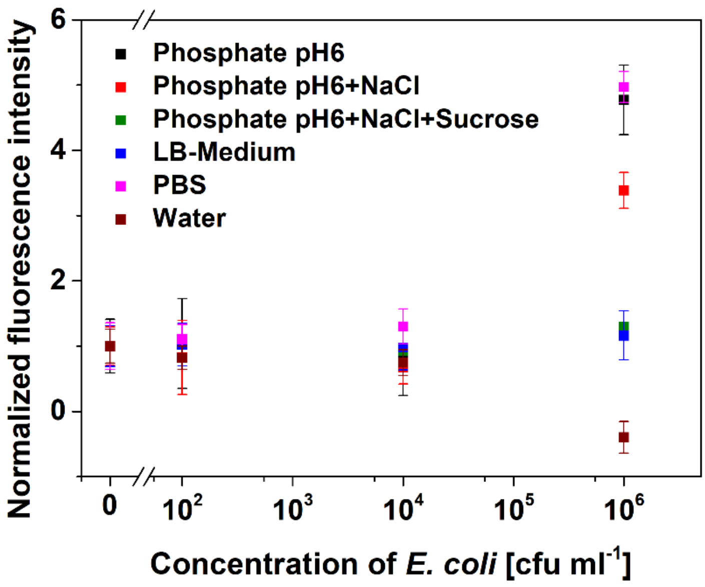

Fig. S5 Microtiter plate assay in different solvents. Fluorescence was read out on a FluostarOPTIMA microplate reader at λ_{exc}=544 nm and λ_{em}=575 nm and a gain of 1500, 3 replicates. Fluorescence intensities were normalized to the blank value

As stated in the main manuscript, changes in the lipid composition were investigated, *i.e.* the EDPPC content of the vesicle membrane was increased to yield a higher positive charge on the liposome surface. We hypothesized that this might be able to enhance the interaction with the bacteria and thus result in lower limits of detection. Figure S6 shows the ζ-potential measurements of the freshly synthesized liposomes. In addition to the 18 mol% (the standard amount of EDPPC in the previous experiments), 30, 40 and 50 mol% of EDPPC were mixed with DPPC. It is clearly visible that the surface potential increases from +15 mv to +25 mV with increasing amount of the cationic phospholipid. It was possible to increase the ζ-potential even more, above +30 mV, when creating a 50:50 mixture with cholesterol and no DPPC at all. Both of the 50 mol% variations were then applied to the centrifugation assay for bacteria detection. Figure S7 shows the dose response curve for both types of liposomes. In

both cases no improvement in the assay sensitivity was observed. Thus, the higher positive surface charge did not improve the interaction but may have led to an increased colloidal stability of the vesicles that rather prevents agglomeration with other particles. Therefore, the standard cationic liposomes with 18 mol% of EDPPC are the better choice.

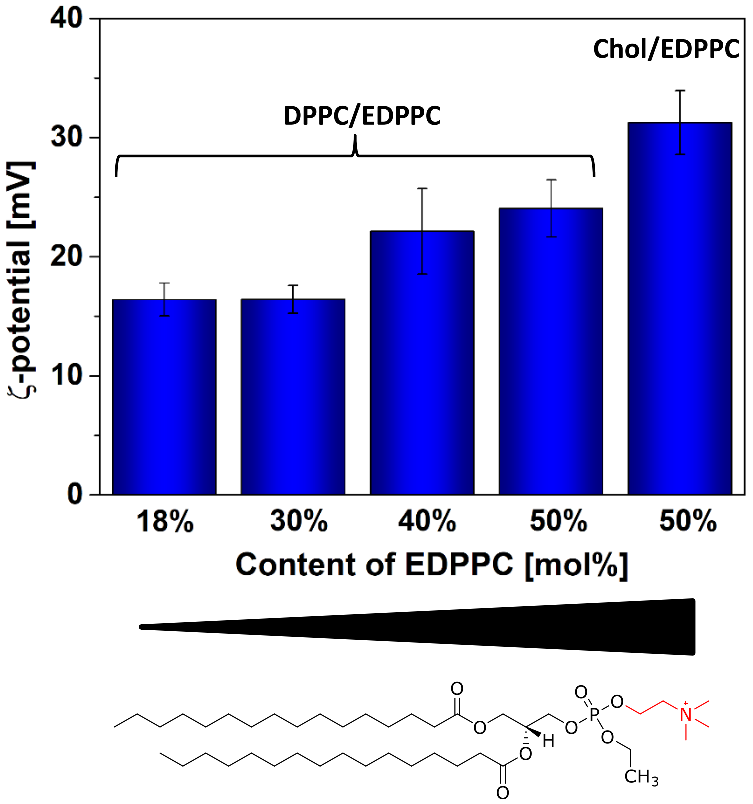

Fig. S6 ζ-potentials of SRB containing liposomes with different mixtures of EDPPC and DPPC and EDPPC and cholesterol, 4 replicates

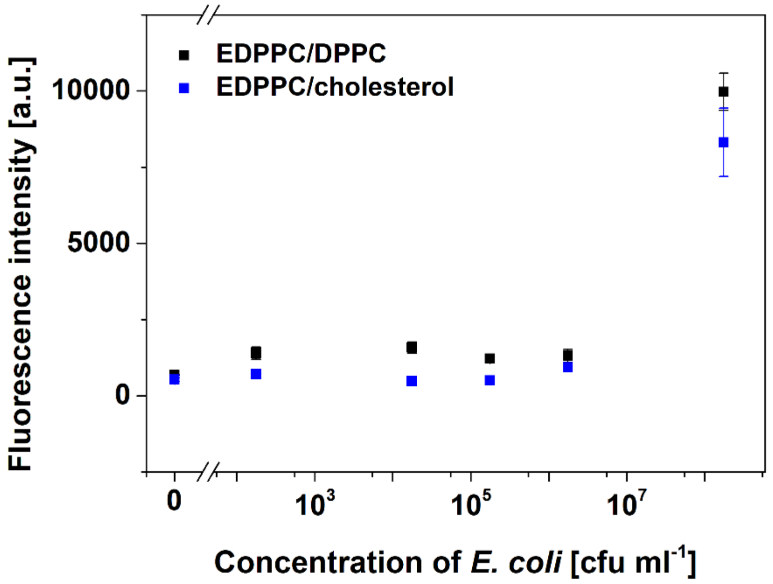

Fig. S7 Centrifugation assay with SRB encapsulating liposomes either composed of EDPPC/DPPC (1:1) or EDPPC/cholesterol (1:1). Fluorescence was read out on a FluostarOPTIMA microplate reader at λ_{ex}=544 nm and λ_{em}=575 nm and a gain of 1200, 4 replicates (error bars are provided, but are in some instances smaller than the chosen size of the symbols)

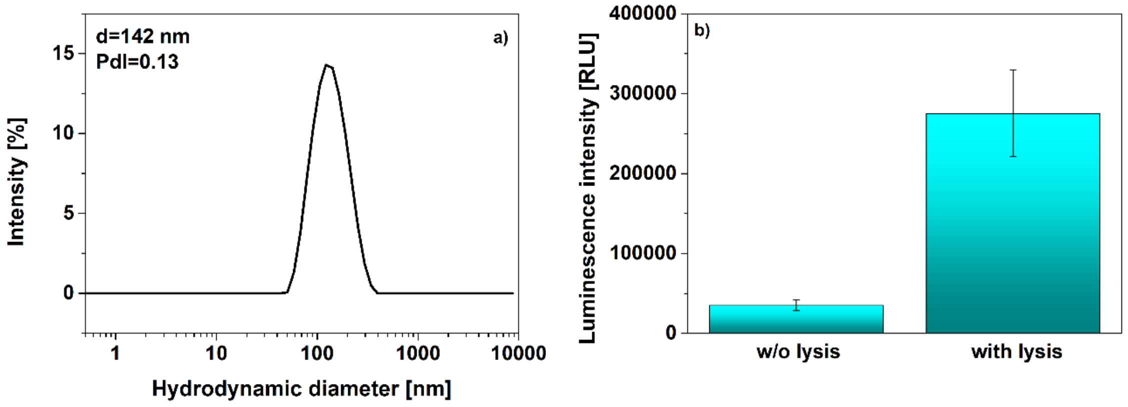

Fig. S8 a) DLS spectrum of cationic m-carboxy-luminol containing liposomes in HSS buffer, n=3. b) Chemiluminescence intensities of intact and lysed cationic m-carboxy-luminol liposomes in carbonate buffer (0.1 M, pH 10.5), 4 replicates. Chemiluminescence measurements were conducted on a BioTek microplate reader with Hemin and H_2O_2 as co-reactants, a read height of 6 mm and a gain of 60

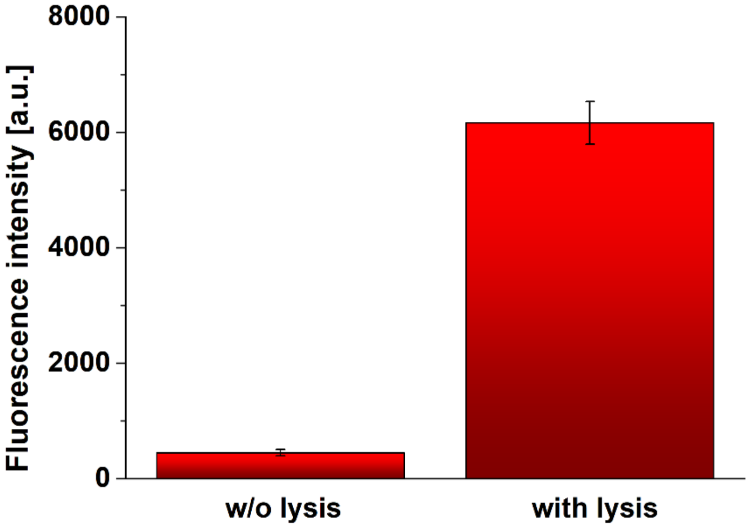

Fig. S9 Fluorescence intensities of intact and lysed cationic SRB liposomes in HSS buffer (10 mM HEPES, 200 mM NaCl, 200 mM sucrose, 0.01% NaN_3, pH 7.5). Fluorescence was read out on a FluostarOPTIMA microplate reader at λ_{exc}=544 nm and λ_{em}=575 nm and a gain of 1200, 3 replicates

2. Methods

Buffer optimizations for bacteria assay on Poly-L-Lysine-coated microtiter plate

The *E. coli* stock solution diluted in different solutions to concentrations between 0 and 10^6 colony forming units (cfu) ml^{-1}: water, LB-medium, PBS, phosphate buffer (10 mM, pH 6), phosphate-NaCl buffer (10 mM phosphate, 200 mM NaCl, pH 6), phosphate-NaCl-sucrose buffer (10 mM phosphate, 200 mM NaCl, 200 mM sucrose, pH 6). The dispersions were then incubated in the wells of a poly-L-lysine coated microtiter plate at room temperature for 1 h. After washing twice with PBS buffer cationic liposomes (50 µM diluted in the respective buffer or medium, 100 µL/well) were added and incubated for 1 h at room temperature. The wells were then washed twice with the respective buffer or medium (200 µl/well). For fluorescence analysis the respective buffer or medium (100 µl/well) was added and the fluorescence read out with a FLUOStar OPTIMA microplate reader (BMG Labtech) (λ_{exc}=544 nm and λ_{em}=575 nm, gain: 1500) before (background) and after lysis of the liposomes with n-Octyl-β-D-glucopyranoside solution (OG, 300 mM diluted in the respective medium or buffer, 10 µl/well). Three individual measurements of each *E. coli* dilution were made. For analysis, the background fluorescence was subtracted from the intensity after lysis. The graph is shown in Figure S5.

References

1. Edwards KA, Meyers KJ, Leonard B, Baeumner AJ. Superior performance of liposomes over enzymatic amplification in a high-throughput assay for myoglobin in human serum. Anal. Bioanal. Chem. 2013; https://doi.org/10.1007/s00216-013-6807-3
